# The obesity-induced transcriptional regulator TRIP-Br2 mediates visceral fat endoplasmic reticulum stress-induced inflammation

**DOI:** 10.1038/ncomms11378

**Published:** 2016-04-25

**Authors:** Guifen Qiang, Hyerim Whang Kong, Difeng Fang, Maximilian McCann, Xiuying Yang, Guanhua Du, Matthias Blüher, Jinfang Zhu, Chong Wee Liew

**Affiliations:** 1 Department of Physiology and Biophysics, College of Medicine, University of Illinois at Chicago, 835 S. Wolcott Avenue, M/C901, MSB E-202, Chicago, 60612 Illinois, USA; 2 Laboratory of Immunology, National Institute of Allergy and Infectious Diseases, NIH, 10 Center Drive, Bethesda, 20892 Maryland, USA; 3 State Key Laboratory of Bioactive Substance and Function of Natural Medicines, Institute of Materia Medica, Chinese Academy of Medical Sciences and Peking Union Medical College, Beijing 100050, China; 4 Department of Medicine, University of Leipzig, Liebigstrasse 18, Leipzig 04103, Germany

## Abstract

The intimate link between location of fat accumulation and metabolic disease risk and depot-specific differences is well established, but how these differences between depots are regulated at the molecular level remains largely unclear. Here we show that TRIP-Br2 mediates endoplasmic reticulum (ER) stress-induced inflammatory responses in visceral fat. Using *in vitro*, *ex vivo* and *in vivo* approaches, we demonstrate that obesity-induced circulating factors upregulate TRIP-Br2 specifically in visceral fat via the ER stress pathway. We find that ablation of TRIP-Br2 ameliorates both chemical and physiological ER stress-induced inflammatory and acute phase response in adipocytes, leading to lower circulating levels of inflammatory cytokines. Using promoter assays, as well as molecular and pharmacological experiments, we show that the transcription factor GATA3 is responsible for the ER stress-induced TRIP-Br2 expression in visceral fat. Taken together, our study identifies molecular regulators of inflammatory response in visceral fat that—given that these pathways are conserved in humans—might serve as potential therapeutic targets in obesity.

## Abstract (web summary)

Visceral and subcutaneous fat are associated with different metabolic risk, but mediators of such depot specific effects are not very well known. Here the authors identify the transcriptional regulator, TRIP-Br2, as a regulator of endoplasmic reticulum (ER) stress-induced inflammatory responses specifically in visceral fat.

## Graphical abstract

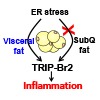

Obesity is a worldwide health problem that impacts the risk and progression of many diseases including diabetes, cardiovascular disease, hyperlipidaemia and certain types of cancer^1^. In humans, the anatomical patterns of fat accumulation during obesity have gained considerable interest because of their association with differential metabolic risks^2^. Accumulation of visceral fat is associated with high risk of metabolic disease and increased mortality, even in those with a normal body mass index. In contrast, increase in subcutaneous (SQ) fat, especially in the gluteofemoral region, confers little or no risk and may even be protective^3^,^4^,^5^,^6^,^7^. Depot-specific differences, including insulin sensitivity, adipogenesis, adipokines secretion and beige adipocyte induction potential, are well characterized, but the mechanisms responsible for these differences are not well understood^8^,^9^.

Recent studies have demonstrated intrinsic gene expression differences between visceral and SQ fat depots^10^,^11^,^12^,^13^. Change in expression of Shox2, a gene expressed in SQ but not visceral fat, affects SQ adipose tissue functions^14^, confirming the importance of differentially expressed genes in different fat depots. However, visceral fat-specific regulators are not well studied. Considering the metabolic risks associated with visceral fat, identification of key visceral fat-specific regulators are likely to have high therapeutic potential to combat obesity-induced metabolic diseases.

Previously, we showed that a transcriptional regulator, TRIP-Br2 (also known as SERTAD2), is specifically upregulated in visceral but not SQ fat in obese men and women. Further, we have demonstrated that ablation of TRIP-Br2 protects mice from obesity and associated metabolic dysfunction^15^. Our previous study demonstrated a role for TRIP-Br2 in adipocyte biology and energy metabolism. Owing to the striking conservation of visceral fat-specific regulation of TRIP-Br2 expression in human and mouse white adipose tissues as well as the metabolic protective phenotype of the knockout (KO) mouse, we hypothesized that TRIP-Br2 plays a role in the regulation of differential metabolic risks between different white fat depots during obesity, especially in visceral adipocytes. Therefore, understanding the upstream regulatory pathway of TRIP-Br2 in visceral fat and its visceral fat-specific functions could yield critical information on the mechanisms leading to visceral fat-induced metabolic risks.

In our present study, we found that expression of TRIP-Br2 in visceral fat is specifically regulated by obesity-induced inflammatory cytokines and fatty acids via the endoplasmic reticulum (ER) stress pathway. We found that modulation of TRIP-Br2 expression significantly altered the expression of visceral adipocyte inflammatory adipokines and acute phase response gene. In addition, we observed that transcription factor GATA3 is similarly regulated in visceral fat upon physiological or pharmacological ER stress induction and inhibition of GATA3 abolished visceral adipocyte ER stress-induced TRIP-Br2 expression.

## Results

### Adipose and macrophages secreted factors modulate TRIP-Br2

**Figure 1:**
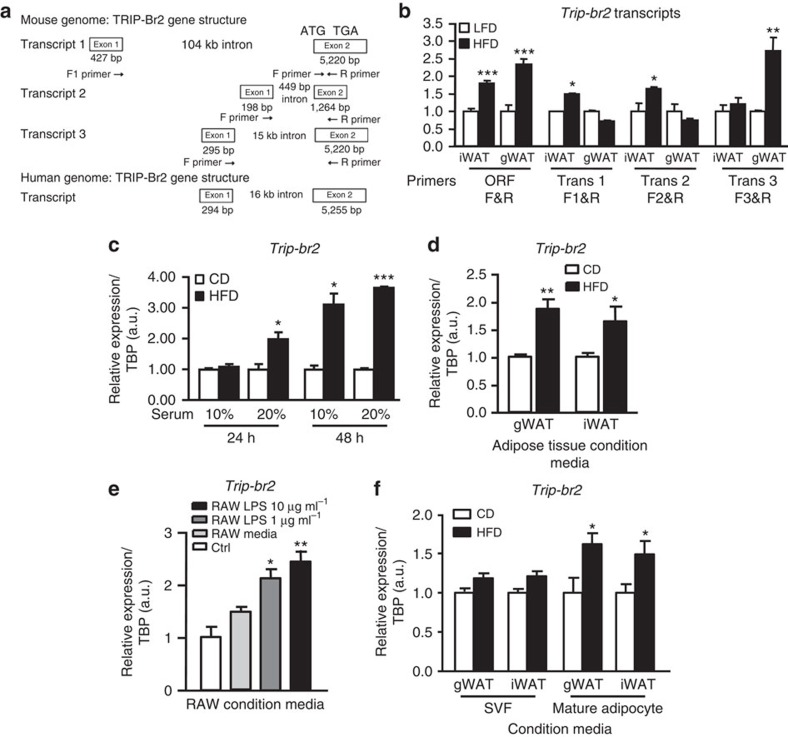
Adipocyte TRIP-Br2 expression is regulated by adipose- and macrophage-secreted factors. (**a**) Schematic depicting exon-intron structure of TRIP-Br2 isoforms in mouse and human genomes. qPCR analysis of TRIP-Br2 gene expression in (**b**) gonadal (gWAT) or inguinal (iWAT) adipose tissues after 12 weeks (wk) of low-fat diet (LFD) or high-fat diet (HFD; *n*=6 per group replicated twice); (**c**) 3T3-L1 adipocytes treated with serum from either chow diet (CD) or HFD mice for 24 or 48 h (*n*=4 per group replicated thrice); (**d**) 3T3-L1adipocytes treated with conditioned media from adipose tissues harvested from mice fed with 12 wk of CD or HFD (*n*=5 per group replicated twice); (**e**) 3T3-L1 adipocytes treated with RAW-conditioned media with or without LPS (1, 10 μg ml^−1^) stimulation for 24 h (*n*=5 per group replicated twice). ANOVA analysis. (**f**) 3T3-L1 adipocytes treated with conditioned media of isolated stromal-vascular-fraction (SVF) or mature adipocytes from CD or HFD-fed mice (*n*=5 per group). All qPCR data are normalized to TATA box-binding protein (TBP) and presented as mean±s.e.m. Two-tailed Student's *t*-test or ANOVA, *P<0.05; **P<0.01; ***P<0.001.

Our previous study demonstrated that TRIP-Br2 is specifically upregulated in visceral but not SQ fat in obese humans and ablation of TRIP-Br2 protects mice from diet-induced obesity and metabolic dysfunction^15^. However, we also observed that TRIP-Br2 expression was similarly regulated in both gonadal (gWAT) and inguinal (iWAT) white adipose tissues in obese mice^15^. This observation limits the use of the mouse model to examine visceral fat-specific TRIP-Br2 regulation. More recently, we discovered that, unlike in humans, there are three TRIP-Br2 isoforms (encoded by a different Exon 1 but coding for the same protein) in mice (Fig. 1a). We noted that transcript 3 is structurally similar to the human transcript (Fig. 1a) and has highest relative expression in both iWAT and gWAT (Supplementary Fig. 1a) compared with transcripts 1 and 2. Interestingly, we observed that transcript 3 is specifically upregulated in gWAT in the high-fat diet (HFD) fed mice (Fig. 1b). These results recapitulate our observation in our human cohorts. Therefore, understanding the visceral fat-specific regulation of the mouse *Trip-br2* transcript 3 might provide mechanistic insight on how TRIP-Br2 is specifically regulated in visceral fat in humans.

To identify upstream factors leading to the increased expression of TRIP-Br2 in visceral fat during obesity, we incubated differentiated 3T3-L1 adipocytes with serum obtained from mice fed either chow diet (CD) or 12 weeks of HFD and observed that HFD serum significantly upregulated *Trip-br2* expression in adipocytes compared with CD serum (Fig. 1c). To determine the source of the upstream factor, we incubated differentiated adipocytes with conditioned media from adipose tissues harvested from CD- or HFD-fed mice. Interestingly, we observed that conditioned media from HFD adipose tissues also significantly upregulated *Trip-br2* expression in 3T3-L1 adipocytes (Fig. 1d). As HFD induces accumulation and activation of immune cells, most prominently the macrophages^16^,^17^, in the adipose tissues, to determine whether macrophages play a role in TRIP-Br2 regulation, we treated differentiated adipocytes with RAW (macrophage cell line)-conditioned media with or without lipopolysaccharides (LPS) stimulation. Similarly, we observed that RAW-conditioned media, especially RAW cells stimulated with LPS but not LPS alone, significantly upregulated *Trip-br2* expression in adipocytes (Fig. 1e and Supplementary Fig. 1b). To confirm our findings and determine the contribution of mature adipocytes and stromal-vascular fraction (SVF) cells on *Trip-br2* induction, we isolated mature adipocytes and SVF from either CD- or HFD-fed mice to obtain respective conditioned media. Interestingly, we observed that the conditioned media from mature adipocytes, but not the SVF isolated from the HFD mice, stimulated *Trip-br2* expression in 3T3-L1 adipocytes (Fig. 1f). These data at first glance seem contradictory to our earlier experiment (Fig. 1e) but SVF cells (that is, macrophages) isolated from their *in vivo* environment might have lost their stimuli resulting in decreased proinflammatory cytokines secretion. If this is the case, these data are in line with our RAW cells study as unstimulated RAW cells did not significantly upregulate *Trip-br2* expression in differentiated adipocytes (Fig. 1e). In addition, we also observed that during the process of SVF isolation, compared with the intact adipose tissues, significant amount of macrophages, as examined by the F4/80 expression, were lost (Supplementary Fig. 1c), potentially causing low level of cytokines in the SVF-conditioned media. Taken together, our results suggest that TRIP-Br2 is regulated by circulating factors secreted by HFD-fed adipose tissues and activated macrophages.

### Adipocyte TRIP-Br2 is regulated by the ER stress pathway

**Figure 2:**
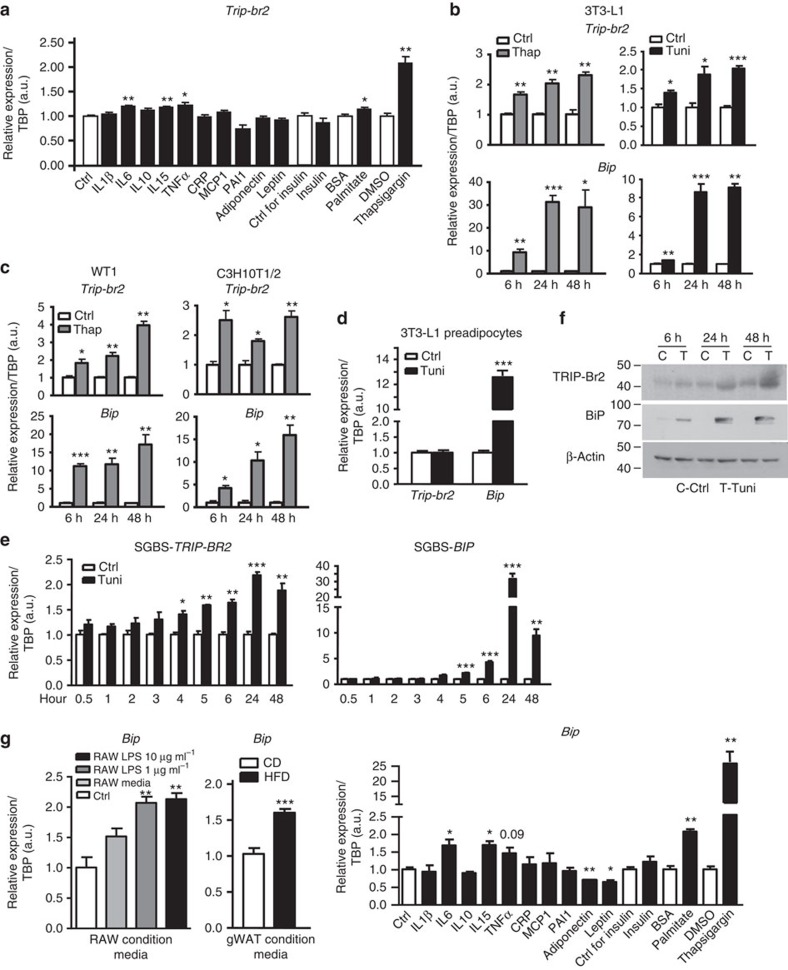
Obesity-induced circulating factor converged on ER stress pathway to regulate TRIP-Br2 expression *in vitro*. qPCR analysis of TRIP-Br2 or ER stress marker (BiP) gene expression in (**a**) 3T3-L1 adipocytes after 24 h treatment with control (Ctrl) or compound indicated (*n*=4 per group replicated twice); (**b**) 3T3-L1 adipocytes after 6, 24 or 48 h treatment with vehicle, thapsigargin (Thap; 0.1 μM) or tunicamycin (Tuni; 1 μg ml^−1^; *n*=3 per group replicated twice); (**c**) WT1 or C3H10T1/2 adipocytes after treatment with vehicle or Thap (0.1 μM) for 6, 24 or 48 h (*n*=3 per group replicated twice); (**d**) 3T3-L1 preadipocytes treated with vehicle or Tuni (1 μg ml^−1^) for 24 h (*n*=3 per group replicated twice); (**e**) human SGBS adipocytes treated with vehicle or Tuni (1 μg ml^−1^) for indicated time points (*n*=3 per group replicated twice). (**f**) Western blot analysis for TRIP-Br2, BiP or β-actin (loading Ctrl) protein in 3T3-L1 adipocytes treated with vehicle (control, C) or Tuni (1 μg ml^−1^, T) for indicated time points. (**g**) qPCR analysis of BiP gene expression in 3T3-L1 adipocytes treated with RAW- or HFD-fed gWAT-conditioned media for 24 h (*n*=5 per group replicated twice) or 3T3-L1 adipocytes after 24 h treatment with Ctrl or compound indicated (*n*=4 per group replicated twice). All qPCR data are normalized to TATA box-binding protein (TBP) and presented as mean±s.e.m. Two-tailed Student's *t*-test or ANOVA, *P<0.05; **P<0.01; ***P<0.001. C, control; T, Tunicamycin.

To identify the potential TRIP-Br2 inducer, we treated differentiated 3T3-L1 adipocytes with factors known to be elevated during obesity, including cytokines, adipokines and fatty acids, as well as the ER stress-inducing agent thapsigargin (Supplementary Table 1). We observed that the cytokines interleukin (IL) 6, IL15 and tumour necrosis factor α (TNFα) and the fatty acid palmitate could significantly increase *Trip-br2* expression individually, although their individual effects are relatively small, whereas thapsigargin significantly upregulated *Trip-br2* expression to the level observed in adipose tissues from HFD-fed mice^15^, a physiological ER stress model (Fig. 2a). To confirm the role of ER stress on TRIP-Br2 expression in adipocytes, we first treated the 3T3-L1 adipocytes with thapsigargin or tunicamycin, another commonly used ER stress chemical inducer. Our results showed that ER stress regulates *Trip-br2* in 3T3-L1 adipocytes in a time-dependent manner in line with the upregulation of ER stress markers (Fig. 2b). To further confirm the role of ER stress, we treated adipocytes differentiated from other preadipocyte cell lines and observed consistent results (Fig. 2c). Further, we showed that ER stress-induced *Trip-br2* expression is absent in undifferentiated preadipocytes, confirming its specificity (Fig. 2d).

Consistently, we also observed that *TRIP-BR2* is similarly regulated in human adipocytes differentiated from the preadipocyte cell line (SGBS) in a time-dependent manner^18^ upon ER stress induction (Fig. 2e). Using western blotting analysis, we confirmed that the transcriptional regulation of *Trip-br2* was accompanied by TRIP-Br2 protein upregulation (Fig. 2f). Finally, to determine the role of ER stress on *Trip-br2* expression in adipocytes treated with adipose- or macrophage-conditioned media (Fig. 1) as well as in cells treated with individual adipokines or fatty acid (Fig. 2a), we examined the expression of ER stress markers. In line with our data obtained so far, we observed that *Trip-br2* upregulation is concomitant with the upregulation of ER stress in the conditioned media-treated adipocytes as well as adipocytes treated with IL6, IL15 and palmitate (Fig. 2g). Taken together, our current data suggest that secreted factors from HFD-fed adipose tissues and activated macrophages such as inflammatory cytokines and fatty acids are potential upstream regulators, but ER stress might be the converging pathway for TRIP-Br2 regulation during obesity.

### ER stress regulates human and mouse visceral fat TRIP-Br2

**Figure 3:**
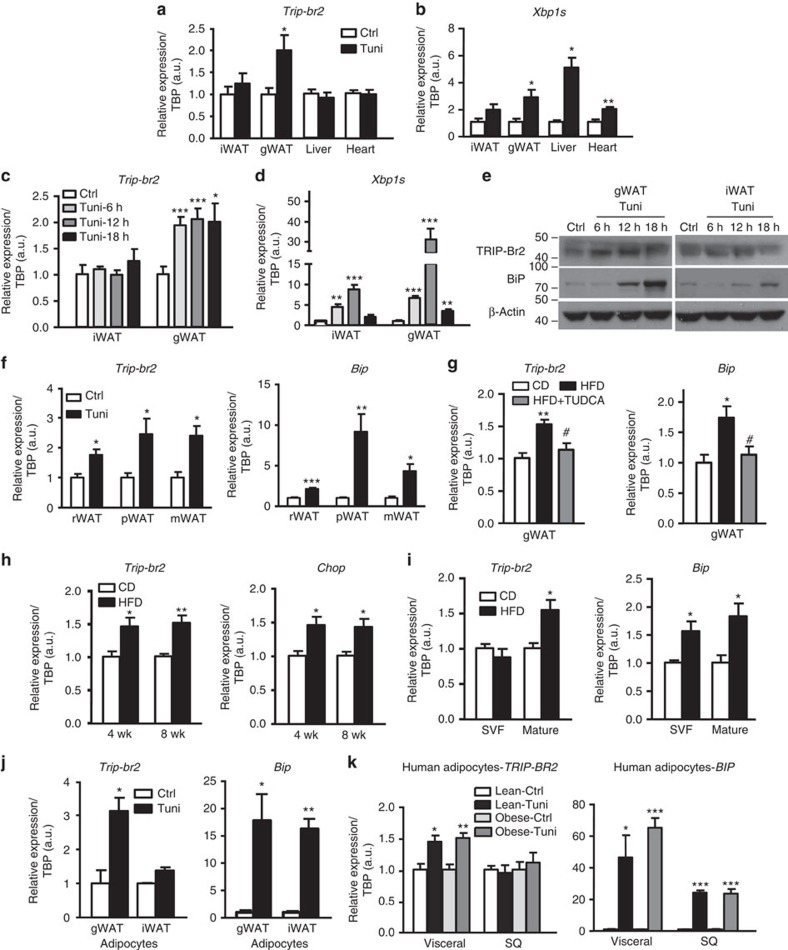
Both chemically- and HFD-induced ER stress promote TRIP-Br2 expression specifically in visceral fat. qPCR analysis of TRIP-Br2 or ER stress marker (XBP1s) gene expression in (**a**,**b**) tissues harvested from mice 18 h after intraperitoneal (i.p.) injection with vehicle (Ctrl, control) or tunicamycin (Tuni; 2.5 mg kg^−1^; *n*=5 per group replicated thrice); (**c**,**d**) iWAT or gWAT from mice 6, 12 or 18 h after IP injection with vehicle or Tuni (2.5 mg kg^−1^; *n*=5 per group replicated twice). (**e**) Western blot analysis of TRIP-Br2, BiP or β-actin (loading control (Ctrl)) in iWAT or gWAT from mice treated for 6, 12 or 18 h with vehicle or Tuni (2.5 mg kg^−1^, i.p.). qPCR analysis of TRIP-Br2 or ER stress marker gene expression in (**f**) retroperitoneal (rWAT), perirenal (pWAT) or mesenteric (mWAT) adipose tissues after IP injection with vehicle or Tuni (2.5 mg kg^−1^; *n*=5 per group replicated thrice); (**g**) gWAT from mice after 12 weeks of CD, HFD or HFD with TUDCA (250 mg kg^−1^ at 0800 hours and 2000 hours i.p., total 500 mg kg^−1^ for 15 days; *n*=5 per group); (**h**) gWAT from mice after 4 or 8 wk of CD or HFD (*n*=5 per group); (**i**) SVF or mature adipocytes from mice after 12 wk of CD or HFD (*n*=5 per group replicated twice); (**j**) gWAT or iWAT adipocytes differentiated from immortalized gWAT or iWAT preadipocytes after 24 h of vehicle or Tuni (1 μg ml^−1^) treatment (*n*=3 replicated twice); (**k**) human adipocytes differentiated from immortalized preadipocytes from lean or obese patients treated with vehicle or Tuni (1 μg ml^−1^) for 24 h (*n*=4 replicated twice). All qPCR data are normalized to TATA box-binding protein (TBP) and presented as mean±s.e.m. Two-tailed Student's *t*-test or ANOVA, *P<0.05; **P<0.01; ***P<0.001 (versus Ctrl or CD); ^#^*P*<0.05 (versus HFD).

To determine whether ER stress induction also induces TRIP-Br2 expression *in vivo*, we injected C57BL/6 (wild-type, WT) mice with vehicle or tunicamycin. Tissues were harvested for analysis 18 h after treatment. Interestingly, we observed that tunicamycin significantly and specifically upregulated *Trip-br2* in gWAT but not in other metabolic organs including iWAT, liver and heart (Fig. 3a). Failure of *Trip-br2* regulation in the liver and the heart despite successful induction of ER stress, as shown by ER stress markers (Fig. 3b, Supplementary Fig. 2a), suggests that *Trip-br2* is not regulated by ER stress in the liver and the heart, consistent with our previous observation in the HFD-fed mice^15^.

As iWAT failed to show any ER stress response after 18 h of tunicamycin treatment, to find out whether SQ fat is resistant to ER stress induction by tunicamycin or has a different response kinetic, we treated WT mice for different time points. Our experiments confirmed that ER stress indeed plays a specific regulatory role on *Trip-br2* regulation in gWAT, because despite successful ER stress induction, we still failed to detect changes in *Trip-br2* expression in the iWAT (Fig. 3c,d and Supplementary Fig. 2b). Increased TRIP-Br2 protein level was later confirmed in gWAT using western blot analysis (Fig. 3e). In addition to gWAT, we observed that *Trip-br2* expression was also upregulated in other visceral fat depots, for example, retroperitoneal, perirenal and mesenteric fat (Fig. 3f), confirming its visceral fat specificity.

As tunicamycin is a chemical ER stress inducer, to determine whether TRIP-Br2 is regulated by physiological ER stress and whether gWAT TRIP-Br2 induction by HFD feeding is actually mediated by ER stress, we treated HFD-fed mice with the ER chaperone tauroursodeoxycholic acid (TUDCA)^19^. Our data showed that TUDCA treatment ameliorated HFD-induced ER stress and at the same time prevented the induction of gWAT *Trip-br2* expression (Fig. 3g and Supplementary Fig. 2c). Further, we also found that even a shorter period (4 or 8 weeks) of HFD was enough to induce ER stress and *Trip-br2* expression in gWAT (Fig. 3h and Supplementary Fig. 2d), confirming the link between gWAT ER stress and TRIP-Br2 regulation. In addition, we also determined that the upregulated *Trip-br2* expression by ER stress in gWAT was confined to mature adipocytes but not the preadipocytes (Fig. 3i), consistent with our *in vitro* study (Fig. 2d). To facilitate subsequent mechanistic studies (see below), we established immortalized gWAT and iWAT preadipocyte cell lines from C57BL/6 mice and confirmed their differentiation capacity with differentiation markers (Supplementary Fig. 2e). Consistent with our *in vivo* data, upon tunicamycin treatment, *Trip-br2* was specifically upregulated in gWAT but not iWAT adipocytes (Fig. 3j).

As we have previously observed that TRIP-Br2 is specifically regulated in visceral fat in overweight and obese patients, to determine the role of ER stress in human visceral adipocytes, we treated differentiated visceral or SQ adipocytes derived from lean or obese patients^20^ with tunicamycin (Supplementary Fig. 2f). Consistently, we observed that *TRIP-BR2* was specifically induced by ER stress in visceral adipocytes but not SQ adipocytes despite robust induction of ER stress markers in both adipocytes (Fig. 3k). Taken together, our *in vivo* and *in vitro* data showed that ER stress (both physiologically and chemically induced) activation plays a selective role in the induction of TRIP-Br2 expression in both human and mouse visceral adipocytes.

### TRIP-Br2 mediates gWAT ER stress-induced inflammation

**Figure 4:**
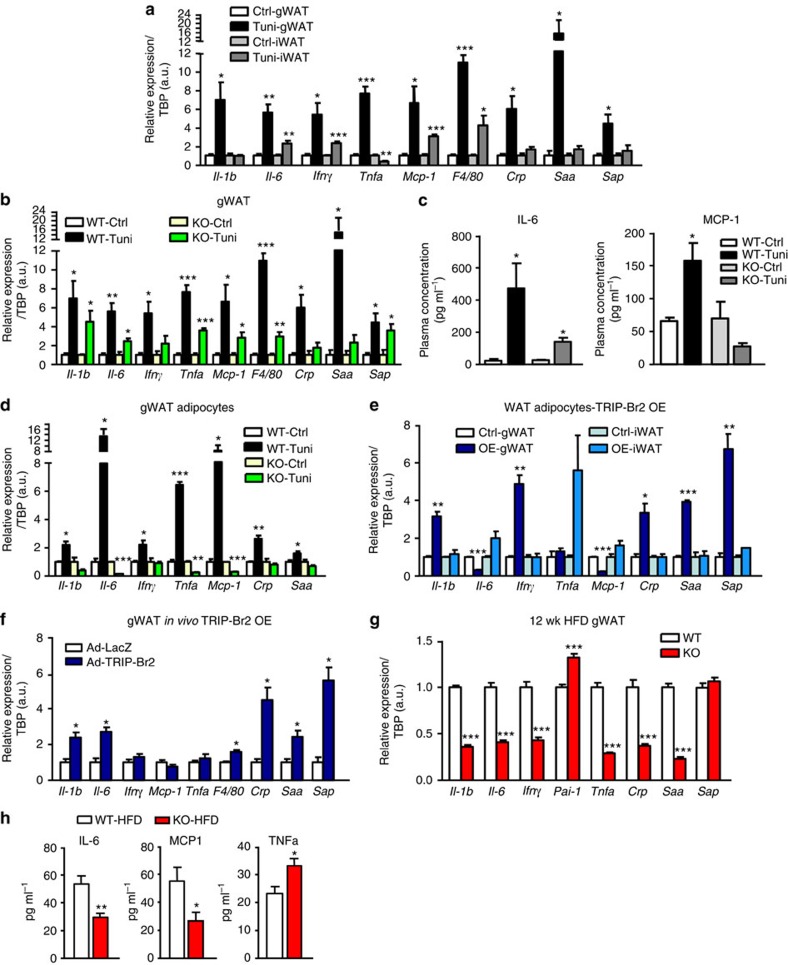
Ablation of TRIP-Br2 abolished ER stress induced-inflammatory and acute phase response in visceral fat. qPCR analysis of inflammation and acute phase reactant markers gene expression in (**a**) gWAT or iWAT fat from C57BL/6 mice treated with vehicle or tunicamycin (Tuni; 2.5 mg kg^−1^, i.p.) for 18 h (*n*=5 per group replicated twice). (**b**) gWAT from TRIP-Br2 WT or KO mice after 18 h of vehicle or tunicamycin (2.5 mg kg^−1^, i.p.) treatment (*n*=5 per group replicated twice). (**c**) Plasma cytokine levels in TRIP-Br2 WT or KO mice after 18 h of vehicle or Tuni (2.5 mg kg^−1^, i.p.) treatment (*n*=5 per group); qPCR analysis of inflammation and acute phase reactant markers gene expression in (**d**) gWAT adipocytes differentiated from primary WT or KO SVF treated with or without Tuni (1 μg ml^−1^) for 24 h (*n*=3 per group replicated twice); (**e**) WT gWAT or iWAT adipocytes with or without TRIP-Br2 overexpression (*n*=3 per group replicated twice). (**f**) gWAT from WT mice 9 days after injection with Ad-LacZ (*n*=6) or Ad-TRIP-Br2 (*n*=8) into gWAT *in vivo*. (**g**) gWAT from WT or KO mice fed with 12 weeks (wk) of HFD (*n*=6 per group replicated twice). All qPCR data are normalized to TATA box-binding protein (TBP) and presented as mean±s.e.m. (**h**) Plasma cytokine levels in WT or KO mice after 12 wk of HFD (*n*=5 per group). Data presented as mean±s.e.m. Two-tailed Student's *t*-test, *P<0.05; **P<0.01; ***P<0.001. Ctrl, control.

Our previous study demonstrated that TRIP-Br2 plays an important role in lipolysis and thermogenesis in visceral fat^15^. Visceral and SQ fats are known to have differential inflammatory adipokines synthesis and secretion during obesity^8^,^9^, and obesity has been shown to induce ER stress in adipose tissues^21^; however, to the best of our knowledge, it has not been carefully examined whether different white fat depots respond differentially to obesity-induced ER stress and whether the potential differences in ER stress response contributes to the differential metabolic risks observed in visceral and SQ fat during obesity. Therefore, to gain insight, we examined expression of inflammatory and acute phase response markers in gWAT and iWAT from WT mice after tunicamycin treatment. Interestingly, we observed that ER stress differentially regulated expression of inflammatory and acute phase response markers in gWAT and iWAT (Fig. 4a). Unlike in the gWAT, these markers are either not induced or induced to a lower level in the iWAT. As we know that TRIP-Br2 is selectively regulated by ER stress in gWAT, to determine whether TRIP-Br2 plays a role in the regulation of the ER stress-induced differential inflammatory and acute phase responses between gWAT and iWAT, we examined the levels of inflammatory and acute phase response markers in gWAT from the TRIP-Br2 KO and WT mice after tunicamycin treatment. Interestingly, we observed that ablation of TRIP-Br2 significantly reduced or abolished expression of ER stress-induced inflammatory and acute phase response markers including *Il-6*, *Ifnγ*, *Tnfa*, *Mcp-1*, *F4/80*, *Crp* and *Saa* in the KO gWAT but not as effectively in the iWAT (Fig. 4b and Supplementary Fig. 3a), which is not surprising as TRIP-Br2 is not regulated in the iWAT. Consistent with the gene expression data, we also observed that circulating levels of IL6 and MCP-1 were upregulated in WT mice treated with tunicamycin but their levels in KO mice were either attenuated or even lowered (Fig. 4c).

As resident and infiltrating immune cells that similarly secrete inflammatory cytokines, for example, macrophages, are also part of the adipose tissues examined in the experiment, in order to rule out the contribution of immune cells in our observation, we also examined inflammatory and acute phase responses in adipocytes differentiated from isolated primary WT or KO gWAT and iWAT SVF upon ER stress induction. Consistent with our observation in the adipose tissues, ablation of TRIP-Br2 abolished ER stress-induced inflammatory and acute phase responses in visceral but not in SQ adipocytes (Fig. 4d and Supplementary Fig. 3b). Lack of contaminating macrophages in our differentiated adipocytes cultured from isolated preadipocytes was evident by an undetectable level of F4/80. To determine the direct contribution of TRIP-Br2, we performed a reciprocal experiment by forced expression of TRIP-Br2 in visceral or SQ adipocytes. Our results confirmed the role of TRIP-Br2 in *Il-1b*, *Ifnγ*, *Crp*, *Saa* and *Sap* expression in the visceral but not SQ adipocytes (Fig. 4e). To determine the effect of TRIP-Br2 *in vivo*, adenoviruses encoding TRIP-Br2 (Ad-TRIP-Br2) or LacZ (Ad-LacZ) were injected into gWAT of WT mice. We observed that forced expression of TRIP-Br2 induced the expression of inflammatory and acute phase response markers in gWAT (Fig. 4f).

Consistent with previous studies, we also observed significant differences in the expression of inflammatory and acute phase response markers between gWAT and iWAT from HFD-fed WT mice, a physiological ER stress inducer (Supplementary Fig. 3c). To determine whether TRIP-Br2 plays a regulatory role in these differences, we examined the inflammatory and acute phase response markers in both the gWAT and the iWAT of WT or KO mice. Similarly, we observed that ablation of TRIP-Br2 significantly reduced the expression of most of the markers in gWAT and reduced their expression to a lesser extent in iWAT (Fig. 4g and Supplementary Fig. 3d). Accordingly, the KO mice also showed significantly lower circulating IL6, and MCP1 levels compared with WT control after HFD (Fig. 4h). Taken together, our data suggest that TRIP-Br2 is a critical molecular mediator for ER stress-induced inflammatory and acute phase responses in visceral fat. Inhibition of TRIP-Br2 upregulated by obesity could be therapeutically beneficial.

### ER stress regulates gWAT TRIP-Br2 via GATA response element

**Figure 5:**
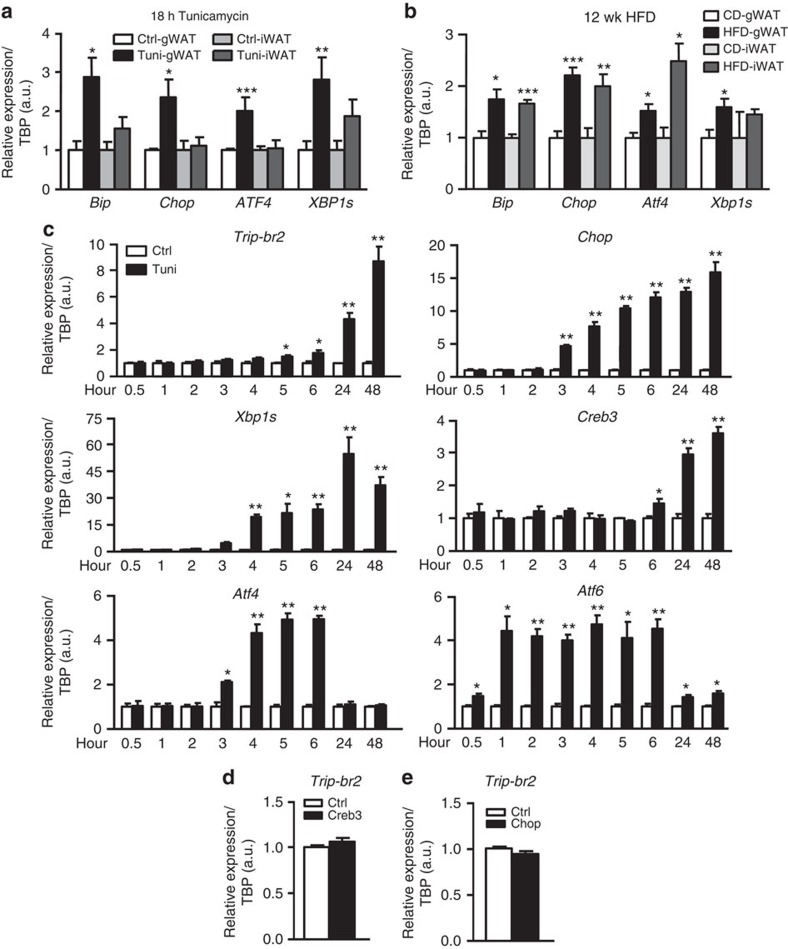
TRIP-Br2 is not regulated by known ER stress-induced transcription factor. qPCR analysis of TRIP-Br2 or ER stress markers gene expression in (**a**) iWAT and gWAT from mice IP injected with vehicle or tunicamycin (2.5 mg kg^−1^) for 18 h (*n*=5 per group replicated twice); (**b**) gWAT and iWAT from mice after 12 wk of CD or HFD (*n*=5 per group replicated twice); (**c**) 3T3-L1 adipocytes treated with vehicle or tunicamycin (1 μg ml^−1^) for indicated time points (*n*=3 per group replicated twice); qPCR analysis of Trip-br2 gene expression in adipocytes stably expressing (**d**) CREB3 (*n*=3 per group) or (**e**) CHOP (*n*=3 per group). All qPCR data are normalized with TATA box-binding protein (TBP) and presented as mean±s.e.m. Two-tailed Student's *t*-test, *P<0.05; **P<0.01; ***P<0.001. Ctrl, control.

As the level of TRIP-Br2 is an important factor in determining the inflammatory and acute phase responses in visceral fat, understanding the pathway regulating visceral fat TRIP-Br2 expression is critical. Even though the ER stress pathway is differentially regulated in gWAT and iWAT by tunicamycin but not with the HFD (Fig. 5a,b), and we also observed that transcription factors, for example, *chop* and *creb3*, showed similar expression time courses in adipocytes (Fig. 5c), it is still possible that one of the known ER stress-induced transcriptional regulators plays a role in regulating TRIP-Br2 expression. However, our experiments showed that ectopic expression of *chop* or *creb3* failed to induce *Trip-br2* expression in differentiated adipocytes (Fig. 5d,e and Supplementary Fig. 4), suggesting involvement of a novel ER stress-induced regulator in visceral fat.

**Figure 6:**
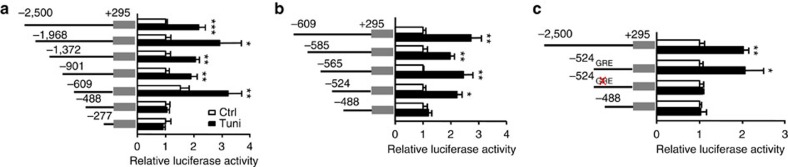
ER stress-induced TRIP-Br2 promoter activity via GATA response element. (**a**–**c**) Luciferase activity (right) of various reporter constructs (left) in differentiated adipocytes transfected with respective constructs and treated with vehicle or tunicamycin (Tuni; 1 μg ml^−1^) for 24 h (*n*=5 per group replicated thrice). All data are normalized with Renilla luciferase activity and presented as mean±s.e.m. Two-tailed Student's *t*-test, *P<0.05; **P<0.01; ***P<0.001. Ctrl, control.

To continue to investigate the underlying molecular mechanisms regulating the gene expression of TRIP-Br2 during ER stress activation, we performed dual-luciferase promoter assays. 2,500 bp (5′ of transcriptional start site) of the promoter region of TRIP-Br2 transcript 3 (TRIP-Br2-003) and the entire exon 1 (295 bp, 5′ untranslated region) were cloned into pGL4 promoter-less luciferase vector. Initial experiments confirmed that the cloned TRIP-Br2-003 promoter construct is responsive to tunicamycin treatment (Fig. 6a), demonstrating that the ER stress-responsive regulatory region is located within the promoter region cloned. TRIP-Br2 promoter was subsequently serially deleted and our data showed that the ER stress-responsive element located within -524:-488 of the promoter region of TRIP-Br2 transcript 3 (Fig. 6a,b). Using *in silico* sequence analyses (Homer, Jasper, TFSEARCH and PROMO)^22^,^23^,^24^,^25^, we identified GATA response element (GRE)-binding and alcohol dehydrogenase regulator 1-binding sites on the TRIP-Br2 promoter. To confirm the functionality, we mutated the GATA response element. Our data showed that mutation of GRE abolished the ER stress-responsive transcriptional activity of the TRIP-Br2 promoter (Fig. 6c).

### ER stress regulates visceral fat TRIP-Br2 via GATA3

**Figure 7:**
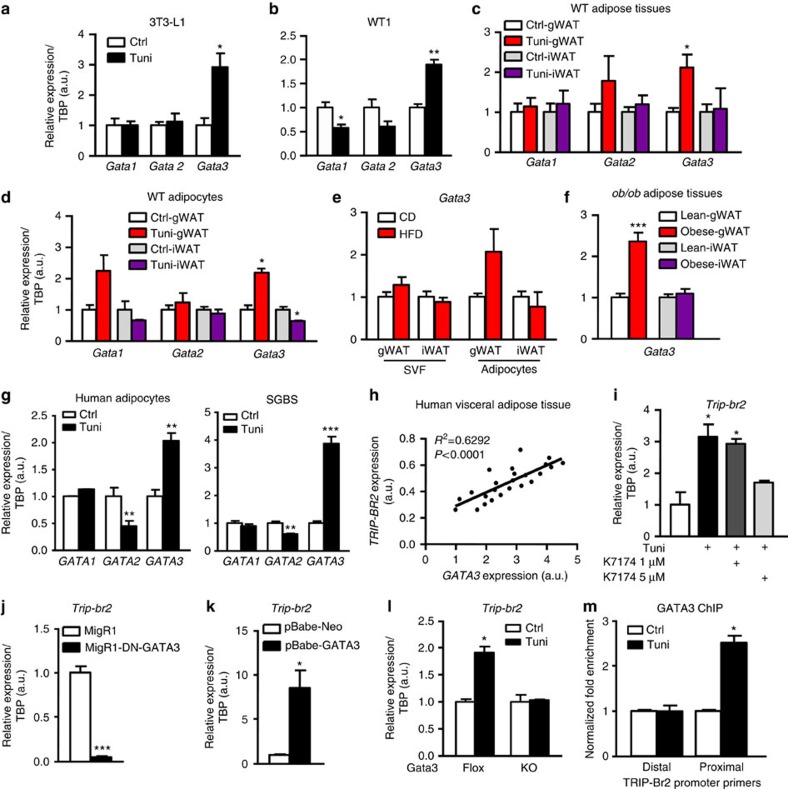
ER stress induces GATA3 expression in visceral adipocytes and leads to TRIP-Br2 upregulation. qPCR analysis of GATA1, GATA2 or GATA3 gene expression in (**a**) 3T3-L1 differentiated adipocytes (*n*=3 per group replicated twice); (**b**) WT1 differentiated adipocytes (*n*=3 per group replicated twice) after 6 h of vehicle or tunicamycin (Tuni; 1 μg ml^−1^) treatment; (**c**) WT gWAT or iWAT (*n*=5 per group replicated twice) 18 h after vehicle or Tuni (2.5 mg kg^−1^, i.p.) injection; (**d**) WT gWAT or iWAT adipocytes (*n*=3 per group) after vehicle or Tuni (1 μg ml^−1^) treatment for 6 h; (**e**) gWAT and iWAT SVF or mature adipocytes after 12 weeks of CD or HFD (*n*=5 per group replicated twice); (**f**) gWAT and iWAT from lean or obese *ob/ob* mice (*n*=6 per group); (**g**) human adipocytes from immortalized human preadipocytes or SGBS cells after vehicle or Tuni (1 μg ml^−1^) treatment for 24 h (*n*=3–4 per group replicated twice). (**h**) Gene expression of human TRIP-Br2 and GATA3 in visceral adipose tissue from human subjects (*n*=24 per group). qPCR analysis of TRIP-Br2 in (**i**) gWAT adipocytes treated with Tuni (1 μg ml^−1^) and/or GATA inhibitor K7174 (1, 5 μM) for 24 h (*n*=3 per group replicated twice); (**j**) gWAT adipocytes infected with control (Ctrl) or DN-GATA3 retrovirus (*n*=3 per group); (**k**) gWAT adipocytes infected with Ctrl or GATA3 retrovirus (*n*=3 per group replicated twice); (**l**) gWAT adipocytes differentiated from GATA3fl/fl-CreERT2 primary SVF with or without tamoxifen (0.5 μM) treatment to induce GATA3 KO (*n*=5 per group). (**m**) qPCR analysis of proximal or distal genomic region of TRIP-Br2 transcript 3 promoter after GATA3 chromatin immunoprecipitation (*n*=3 per group replicated twice). All qPCR data are normalized to TATA box-binding protein (TBP) and presented as mean±s.e.m. Two-tailed Student's *t*-test or ANOVA, *P<0.05; **P<0.01; ***P<0.001.

In addition to a previously known role for GATA2 and GATA3 in adipocytes^26^,^27^, GATA1-3s have been predicted to bind to the GRE site on the TRIP-Br2 promoter by *in silico* software analyses. To identify the GATA isoform responsible for the TRIP-Br2 expression during ER stress induction in visceral fat, we examined expression levels of *Gata1*, *Gata2* and *Gata3* in adipocytes treated with tunicamycin. Consistent with the upregulation of *Trip-br2* upon ER stress induction (Fig. 2b,c), we observed that *Gata3* but not *Gata1* and *Gata2* is upregulated in tunicamycin-treated 3T3-L1 and WT1 adipocytes (Fig. 7a,b), in gWAT but not iWAT (Fig. 7c), and adipocytes differentiated from immortalized gWAT but not iWAT preadipocytes (Fig. 7d). In addition, we also observed that physiological stress induced by HFD- or genetic-induced obesity similarly upregulated *Gata3* expression in gWAT and mature gWAT adipocytes but not in iWAT and iWAT SVF (Fig. 7e,f).

When we examined *GATA* expression in human adipocytes differentiated from immortalized visceral preadipocytes^20^ or SBGS cells, consistent with the mouse model data, we observed that *GATA3* but not *GATA1* and *GATA2* was upregulated upon tunicamycin treatment (Fig. 7g), suggesting species conservation in GATA3 regulation. Further, we also found that *GATA3* expression is positively correlated with *TRIP-BR2* expression in human visceral fat obtained for our previous studies^15^,^28^ (Fig. 7h).

To confirm the direct role of GATA3 on the ER stress-induced TRIP-Br2 expression, we inhibited GATA3 activity using pharmacological inhibitor (K7174) (ref. 29) and found that gWAT adipocytes treated with K7174 showed a dose-dependent inhibition of ER stress-induced *Trip-br2* expression (Fig. 7i). To further confirm that, we expressed dominant negative GATA3 (DN-GATA3) (ref. 30) in differentiated gWAT adipocytes and observed that *Trip-br2* was significantly downregulated (Fig. 7j). In contrast, transient overexpression of GATA3 significantly upregulated *Trip-br2* in gWAT adipocytes (Fig. 7k and Supplementary Fig. 5a). Interestingly, when we expressed GATA3 in iWAT adipocytes, this GATA3 forced expression actually allowed the iWAT adipocyte *Trip-br2* to be inducible by ER stress induction (Supplementary Fig. 5b). This result confirms that GATA3 is required for TRIP-Br2 regulation in adipocytes. To confirm the role of GATA3 in induction of visceral fat TRIP-Br2 expression, we isolated SVF from GATA3fl/fl-CreERT2 mice^31^. Differentiated adipocytes were treated with tamoxifen to induce GATA3 KO before treatment with tunicamycin^31^. Consistent with all our data gathered so far, we observed that ablation of GATA3 in gonadal adipocytes prevented the induction of TRIP-Br2 after tunicamycin treatment (Fig. 7l and Supplementary Fig. 5c).

Finally, using GATA3 chromatin immunoprecipitation, we confirmed binding of GATA3 on TRIP-Br2 promoter upon ER stress induction (Fig. 7m and Supplementary Fig. 5d). Taken together, our studies identified GATA 3 as a novel visceral fat-specific ER stress-induced transcription factor. We showed that GATA3 is responsible for the depot-specific induction of TRIP-Br2 expression by ER stress.

## Discussion

Differences between visceral and SQ fat, especially in the obese state, are well established, but the mechanisms responsible for these differences are not well understood^8^,^9^. Therefore, identification of the regulator mediating the differences could generate potential therapeutic targets to improve systemic metabolic homeostasis. The data presented here clearly demonstrate that in addition to previously identified depot-specific differences including insulin sensitivity, lipid metabolism, adipogenesis and beige adipocyte induction potential, visceral and SQ fat show profound differences in stress-induced inflammatory and acute phase response markers upon challenge with pathophysiological or pharmacological stress stimuli. These differences are in part due to dysregulation of the transcriptional regulator TRIP-Br2 in visceral fat.

In our previous study, we observed that TRIP-Br2 is specifically elevated in overweight and obese human visceral fat but not SQ fat. We then showed that ablation of this regulator protects mice from diet-induced obesity and metabolic dysfunction^15^. However, the mechanism by which TRIP-Br2 is regulated in a depot-specific manner upon nutritional overload remained elusive. As the absence of TRIP-Br2 is metabolically beneficial and our initial studies showed that TRIP-Br2 plays a repressive role on multiple adipocyte functions and processes, it would be critical to dissect the pathway specifically regulating TRIP-Br2 in visceral fat, as this pathway could potentially regulate other metabolic processes in the visceral fat and mediate its metabolic risks during obesity.

In addition to dyslipidaemia, it is well established that obesity also induces a state of chronic systemic low-grade inflammation originating from white adipose tissue. Obesity-induced inflammatory responses involve systemic increases in circulating inflammatory cytokines and acute phase proteins from adipocytes and activated resident and infiltrating immune cells in adipose tissue. Increase in both circulating inflammatory cytokines and fatty acids are known to affect insulin sensitivity and lipid metabolism in multiple metabolic organs including the liver and the muscle. It is believed that chronic local and systemic inflammation are major contributing factors for the metabolic dysfunction observed during obesity^32^. However, recent anti-inflammatory studies have also demonstrated that inflammation might not be critically important for certain obesity-induced co-morbidities^33^,^34^ such as endothelial dysfunction and serum low-density lipoprotein cholesterol levels, suggesting disease- and tissue-specific role for inflammation in obesity pathogenesis.

Our observation that a subset of obesity-induced circulating factors only stimulate the expression of TRIP-Br2 in visceral adipocytes was initially puzzling because it was hard to envision depot specificity as all fat depots are exposed equally to circulating factor. However, our subsequent data demonstrated that the depot specificity is achieved via tissue-specific responses upon exposure to the elevated circulating inflammatory cytokines and fatty acids. This observation is consistent with studies showing that the differences between the different white fat depots are in part contributed by the intrinsic properties of individual adipocytes^10^,^35^.

Later, we showed that the circulating stimulatory signals actually converge on the ER stress pathway to eventually control TRIP-Br2 expression in visceral adipocytes. We know that excess nutrient fluxes through the ER during obesity activate the unfolded protein response in the ER, particularly in the liver and the adipose tissue^21^. Chronic activation of unfolded protein response leads to ER stress, which has been proposed to negatively impact metabolic homeostasis in part through the induction of inflammatory signalling pathways in adipose tissues. However, it is not known whether ER stress-induced inflammation is differentially regulated in different depots. In this study, we showed that ER stress differentially induced inflammatory responses in visceral and SQ fat. Our *in vivo* and *in vitro* data showed that visceral fat tends to be more inflammatory than the SQ fat following ER stressor treatment. To determine the mechanisms behind these differences, based on our observation, we hypothesize that TRIP-Br2 could be a molecular mediator of the inflammatory-prone nature of visceral fat. Indeed, we observed that ablation of TRIP-Br2 abolished most of the ER stress- and HFD-induced expression of inflammatory and acute phase response markers, suggesting that inhibition of TRIP-Br2 could potentially ameliorate obesity-induced visceral fat inflammation.

During the process of examining the effect of TRIP-Br2 KO on the levels of proinflammatory cytokines and acute phase reactants, we observed that the ER stress-induced inflammatory responses between the WT SQ fat and the KO visceral fat are strikingly similar, suggesting that ablation of TRIP-Br2 attenuates visceral fat inflammatory responses to the levels of SQ fat responses. This observation is potentially important because unlike visceral fat, SQ fat is known to play a more protective role in the metabolic homeostasis during obesity; consequently, finding a strategy to convert visceral fat would be clinically relevant and worth future follow-up studies. In addition, we also found that a subset of the cytokines including IL6, TNFα and MCP1 were actually downregulated in the SQ and visceral fat from the TRIP-Br2 KO mice (Fig. 4d and Supplementary Fig. 3b). These findings may seem unexpected as ER stress is known to promote the inflammatory response and IL6, TNFα and MCP1 are generally assumed to be proinflammatory. However, recent studies have provided strong supporting evidence that the classically known proinflammatory cytokines might play context-dependent beneficial roles in addition to their role in inflammation. In fact, it was demonstrated that certain levels of ‘physiological inflammation' in adipose tissue is actually required for the healthy expansion of fat depots upon excess nutritional challenges^36^,^37^. It is currently unclear what is the role of IL6, TNFα and MCP1 downregulation in SQ fat and the KO visceral fat, but this observation certainly warrants further investigation.

Visceral fat-specific TRIP-Br2 upregulation at first glance is due to a much greater ER stress response in the visceral fat upon tunicamycin treatment. However, this hypothesis was later proven to be incorrect as TRIP-Br2 upregulation in visceral fat was still evident in adipose tissue harvested from HFD-induced obese mice, even though the ER stress levels were similarly induced by 12 weeks of HFD feeding in both visceral and SQ fat. As the expression time course and our over-expression data did not support the involvement of the commonly known ER stress-induced transcription factors, we hypothesized that TRIP-Br2 is regulated by ER stress in visceral fat via an uncharacterized ER stress-induced transcriptional regulator. Using multiple *in vitro* and *in vivo* assays, we identified a novel visceral fat-specific, ER stress-induced transcription factor, GATA3.

**Figure 8:**
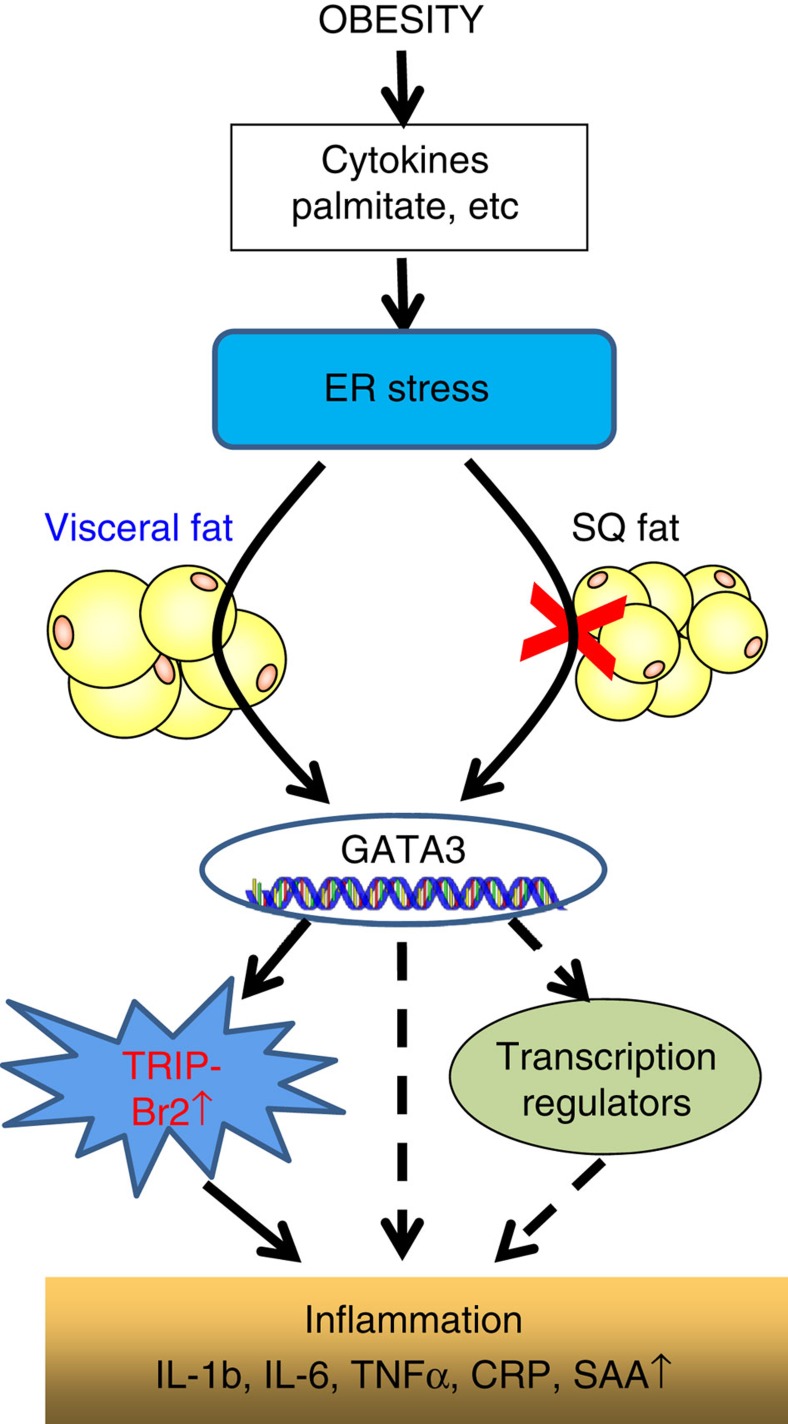
Model for the regulation of visceral fat ER stress in obesity. Induction of TRIP-Br2 and GATA3 during obesity via ER stress is critical for the visceral fat proinflammatory responses.

GATA family transcription factors are generally thought to play important roles in hematopoiesis. These transcription factors have not been studied extensively in adipocytes, except limited literature showing that GATA2 and GATA3 play an inhibitory role in adipogenesis^26^,^27^. It is, however, unknown whether the GATA family of transcription factors also has a role in other adipose tissue functions and metabolic processes. Our current study identified a role for GATA3 in metabolic processes, especially after induction of ER stress in visceral fat. Data from our tissue distribution and ChIP experiments suggest that GATA3 expression and binding on the TRIP-Br2 promoter in visceral fat are inducible after ER stress induction. Importantly, this phenomenon is consistently observed in visceral fat from human subjects, where TRIP-Br2 expression is positively correlated with GATA3 expression. So far, our data position GATA3 between the ER stress pathway and TRIP-Br2 to regulate adipose tissues, particularly the inflammatory response during obesity (Fig. 8). Our current data firmly support previously described functional differences between visceral and SQ fat. Whether GATA3 is the molecular switch that differentiates visceral and SQ fat yet to be determined. It will be important to identify the molecular switch that promotes GATA3 expression upon ER stress induction in the visceral fat in future studies and we also plan to determine whether ER stress-induced GATA3 expression and DNA binding could also regulate other transcriptional regulators besides TRIP-Br2 or other metabolic processes in visceral fat (Fig. 8).

Taken together, our study has identified critical molecular regulators that mediate differential inflammatory responses between visceral and SQ fat induced by excess nutritional stress. Conservation of this regulatory pathway in human fat makes them potential therapeutic targets for the treatment of obesity and obesity-related metabolic diseases.

## Methods

### Reagents

Thapsigargin, tunicamycin (composition: A: 9.3%, B: 24.33%, C: 52.35%, D: 12.57%), TUDCA, LPS, palmitate, human insulin, isobutylmethylxanthine (IBMX), dexamethasone, T3, indomethacin, doxycycline, puromycin, G418, tamoxifen and polybrene were from Sigma-Aldrich. K7174 was obtained from MedKoo Biosciences. IL1β, IL6, IL10, IL15, TNFα, CRP, MCP1, PAI-1, adiponectin and leptin were from R&D Research.

### Antibodies

Mouse polyclonal antibodies to TRIP-Br2 were raised against the recombinant protein of human TRIP-Br2 (ref. 15). Anti-BiP (#3177), anti-GATA3 (#5852), anti-β-actin (#4970) antibodies and SimpleChIP Enzymatic Chromatin IP Kit (Magnetic Beads, #9003) were obtained from Cell Signaling.

### Animals

Mice were housed in environmentally controlled conditions with a 12-h light/dark cycle and had free access to standard rodent pellet food and water. The animal protocols were approved by the Institutional Animal Care and Use Committee of University of Illinois at Chicago. Animal care was given in accordance with institutional guidelines. Male C57BL/6J and *ob/ob* mice were obtained from the Jackson Laboratory. Six- or eight-week-old C57BL/6J animals were used in our experiments. Generation of TRIP-Br2 KO mouse line has been described previously^15^. TRIP-Br2 transgenic animals used in this study are on C57BL/6 background. GATA3fl/fl-CreERT2 mice were generated as previously described^31^.

*High-fat diet treatment*. Six-week-old male C57BL/6 mice were fed on a HFD (60% kcal from fat; Research Diet). After 10 weeks of HFD, the HFD+TUDCA group received intraperitoneal injection of TUDCA twice a day (250 mg kg^−1^ at 0800 hours and 2000 hours, total 500 mg kg^−1^ per day) for 15 days. Age-matched HFD group mice were administered with the same volume of vehicle. Age-matched CD control mice were also administered with vehicle.

*Tunicamycin treatment*. Ten-week-old male TRIP-Br2 WT and KO littermates were injected intraperitoneally with tunicamycin (2.5 mg kg^−1^) dissolved in dimethylsulphoxide and diluted with PBS containing 100 mM glucose, or vehicle (PBS with dimethylsulphoxide and glucose).

*In vivo adenovirus*. Anaesthetized male C57BL/6 mice were injected with Ad-TRIP-Br2 or Ad-LacZ (1.5 × 10^8^ p.f.u. per site; *n*=5) into gonadal adipose tissue as described previously^38^. Animals were monitored daily and they were killed 9 days after injection.

### Plasmids and virus

Open reading frame (ORF) of GATA3 was subcloned from pBabe-Puro-GATA3 (Addgene, #1286) into pBabe-Neo with PCR. Mouse TRIP-Br2 promoters and the respective deletion constructs were amplified from NIH3T3 genomic DNA with PCR using oligonucleotides (IDT) and cloned into the pGL4-promoter luciferase vectors (Promega). MigR1 and DN GATA3 plasmids were gifts from Pear and co-workers^30^. TRIP-Br2 was PCR-amplified and cloned into pENT-TRE plasmid and then into pSLIK-Neo lentivirus vector from Fraser and co-workers^39^ using the Gateway System (Invitrogen). All plasmids were sequence verified. Adenoviruses expressing human TRIP-Br2 or LacZ were purchased from Vigene Biosciences or Vectro BioLabs, respectively.

### Cell lines and cell culture

WT1 and C3H10T1/2 cells were provided by Dr Yu-Hua Tseng laboratory, Joslin Diabetes Center^40^,^41^. 3T3-L1 cells were purchased from American Type Culture Collection. All cells were tested for mycoplasmas contamination before used. All cells were grown in DMEM supplemented with 10% FBS, 100 U ml^−1^ penicillin and 100 μg ml^−1^ streptomycin and maintained at 37 °C with 5% CO_2_ atmosphere in a humidified incubator. Human SBGS and preadipocytes were differentiated as previously described^18^,^20^. Mouse preadipocyte cell lines were differentiated with differentiation cocktail (50 nM insulin, 100 nM T3, 0.125 mM Indomethacin, 0.5 mM IBMX and 5 μM dexamethasone in DMEM with 10% FBS) for 2 days before 4 days in medium supplemented with 50 nM insulin and 1 nM T_3_. ER stress was induced in cells by treatment with thapsigargin (0.1 μM) or tunicamycin (1 μg ml^−1^) for the indicated time points.

### Condition medium

For HFD-conditioned medium, gWAT and iWAT from C57BL/6 male mice fed with 12 weeks of CD or HFD were collected and cultured in 2 ml of DMEM medium in 6-well plate at 37 °C, 5% CO_2_ for 24 h. Supernatant was collected and 10% FBS was added before incubation with differentiated adipocytes.

For RAW-conditioned medium, supernatant from RAW cells treated with control or LPS (1 or 10 μg ml^−1^) for 24 h was used.

### Primary stromal-vascular fraction isolation and differentiation

gWAT or iWAT were dissected from 5- to 6-week-old WT, TRIP-Br2 KO or GATA3fl/fl-CreERT2 male mice, minced and digested with collagenase Type 1 (Worthington; 1 mg ml^−1^ in Kreb Ringer buffer with albumin (KRBA) containing 125 mM NaCl, 4.74 mM KCl, 1 mM CaCl_2_, 1.2 mM KH_2_PO_4_, 1.2 mM MgSO_4_, 5 mM NaHCO_3_, 25 mM HEPES (pH 7.4), 3.5% BSA+5.5 mM glucose) for 30–45 min with shaking at 37 °C (ref. 42), then centrifuged at 1,000 r.p.m. for 5 min. The top adipocytes were gently transferred as mature adipocytes and the SVF pellet was washed twice with KRBA and once with DMEM complete medium. Then, the pellet was resuspended in 5 ml of DMEM complete medium and filtered over 40 μm filter adaptor. Filtered SVF was plated onto rat tail collagen-I (Invitrogen)-coated dish. For differentiation, confluent primary preadipocytes were differentiated with 50 nM insulin, 100 nM T_3_, 0.125 mM indomethacin, 0.5 mM IBMX and 5 μM dexamethasone in DMEM/F12 media supplemented with 10% FBS for 2 days, followed by 4 days in medium supplemented with 50 nM insulin and 1 nM T_3_ with media change in between. To induce GATA3 deletion, D4 differentiated adipocyte were treated with 0.5 μM tamoxifen for 48 h.

### Retrovirus production and infection

Phoenix cells (Orbigen) were transfected with pECO and pBABE-Neo, pBABE-Neo-mGATA3, MigR1 or DN-GATA3 plasmid. Twenty-four hours after transfection, cells were changed with fresh medium and transferred to 33 °C incubator with 5% CO_2_. Forty-eight hours later, virus supernatant was collected and filtered with 0.45 μm filter. Immortalized gWAT or iWAT adipocytes at day 6 were infected with pBABE-Neo, pBABE-mGATA3, MigR1 or DN-GATA3 retrovirus supernatant in 2 μg ml^−1^ polybrene. Twenty-four hours after infection, the adipocytes were harvested for RNA and quantitative PCR (qPCR) analysis.

### Reporter assay

On day 2 of differentiation, adipocytes were co-transfected using jetPRIME (Polyplus) with different fragments of TRIP-Br2 reporter constructs along with a promoterless Renilla luciferase construct. Twenty-four hours after transfection, the cells were incubated with tunicamycin (1 μg ml^−1^). Lysates were collected 24 h after treatment, and firefly and *Renilla* luciferase activities were measured with a Dual-Luciferase Reporter System (Promega).

### Chromatin immunoprecipitation assay

Differentiated adipocytes were treated at day 6 with tunicamycin (1 μg ml^−1^) for 24 h. The cells were then fixed by addition of 37% formaldehyde to a final concentration of 1% formaldehyde and incubated at room temperature for 10 min. Crosslinking was stopped by the addition of glycine to a final concentration of 0.125 M. Cells were then scraped, and samples were prepared using SimpleChIP Enzymatic Chromatin IP Kit (Cell Signaling, #9002) according to the manufacturer's protocol. The chromatin fractions were incubated in each case with 10 μg of antibodies to one of the following: GATA3 (Cell Signaling, #5852), mouse RPL30 or normal mouse IgG (both provided by Cell Signaling kit, Cell Signaling) at 4 °C overnight with Magnetic Protein G Beads. After extensive washing and final elution, the product was treated at 65 °C overnight to reverse the crosslinking. Input DNA and immunoprecipitated DNA were purified using a kit column and analysed by qPCR using SYBR Green Supermix (Bio-Rad) with the following sets of primers (both proximal and distal promoter regions): mouse GATA proximal promoter (forward, 5′-CTCCCCCCATTCCCATATCT-3′; reverse, 5′-TGGGTGATGACAAAGACGGC-3'), mouse GATA distal promoter (forward, 5′-GGAAGGTGCCCTGAAGAGAT-3′; reverse, 5′-GTCCTATGGAGGCCAGAGAG-3'). All results were normalized to the respective input values.

### Immortalization

Exponentially grown primary preadipocytes in the 6-cm dish were infected with pBabe-Puro-SV40 Large T-antigen (Addgene) overnight. Cells were then split into 10 cm dishes and allowed for recovery for 24 h before subjecting to puromycin selection (3 μg ml^−1^) for 1 week.

### Stable inducible cell line

Immortalized gWAT or iWAT preadipocytes were infected with pSLIK-TRE-TAP-Neo or pSLIK-TRE-TAP-hTRIP-Br2-Neo. Twenty-four hours after infection, cells were selected with 1,000 ng ml^−1^ of G418 for up to 2 weeks. TAP or TAP-TRIP-Br2 expression was induced by the addition of 1,000 ng ml^−1^ of doxycycline for indicated time.

### RNA extraction and real-time PCR

Total RNA was isolated from tissues and cells with the use of Trizol reagent (Invitrogen) and Direct-zol kit (Zymo). cDNA was prepared from 1 μg of total RNA using the High Capacity cDNA Reverse Transcription Kit (Invitrogen) with random hexamer primers, according to the manufacturer's instructions. The resulting cDNA was diluted tenfold, and a 1.5-μl aliquot was used in a 6-μl PCR reaction (SYBR Green, Bio-Rad) containing primers at a concentration of 300 nM each. PCR reactions were run in triplicate and quantitated using the Applied Biosystems ViiA 7 Real-Time PCR system. Results were normalized to *TATA box-binding protein* (*TBP*) expression and expressed as arbitrary units or fold change. Primer sequences listed in Supplementary Table 2.

### Western blotting

Total cell or tissue lysates (20−50 μg) were subjected to SDS−polyacrylamide gel electrophoresis and blotting was performed as described^43^. Multiple exposures were used to ascertain signal linearity. Images have been cropped for presentation. Uncropped images of western blots are shown in Supplementary Figs 6 and 7.

### Statistical analyses

All data are presented as the mean±s.e.m. (standard error of mean) and were analysed by unpaired two-tailed Student's *t-*test or analysis of variance, as appropriate. *P*<0.05 was considered significant. Studies were performed on two or three independent cohorts and were performed on four to five mice per group unless specified. Samples size was determined using previous experiments on the characterization of the mouse model used in this study. Mice were randomized to treatment in a blinded manner.

## Additional information

**How to cite this article:** Qiang, G. *et al*. The obesity-induced transcriptional regulator TRIP-Br2 mediates visceral fat endoplasmic reticulum stress-induced inflammation. *Nat. Commun.* 7:11378 doi: 10.1038/ncomms11378 (2016).

## Supplementary Material

Supplementary Information Supplementary Figures 1-7 and Supplementary Tables 1-2.
