# Supplementary material for: The obesity-induced transcriptional regulator TRIP-Br2 mediates visceral fat endoplasmic reticulum stress-induced inflammation
Source: Nat Commun. 2016 Apr 25;7:11378. doi: 10.1038/ncomms11378 (PMC4848483; doi:10.1038/ncomms11378)
Supplement: Supplementary Information — Supplementary Figures 1-7 and Supplementary Tables 1-2. [file ncomms11378-s1.pdf]

Supplementary Figure 1

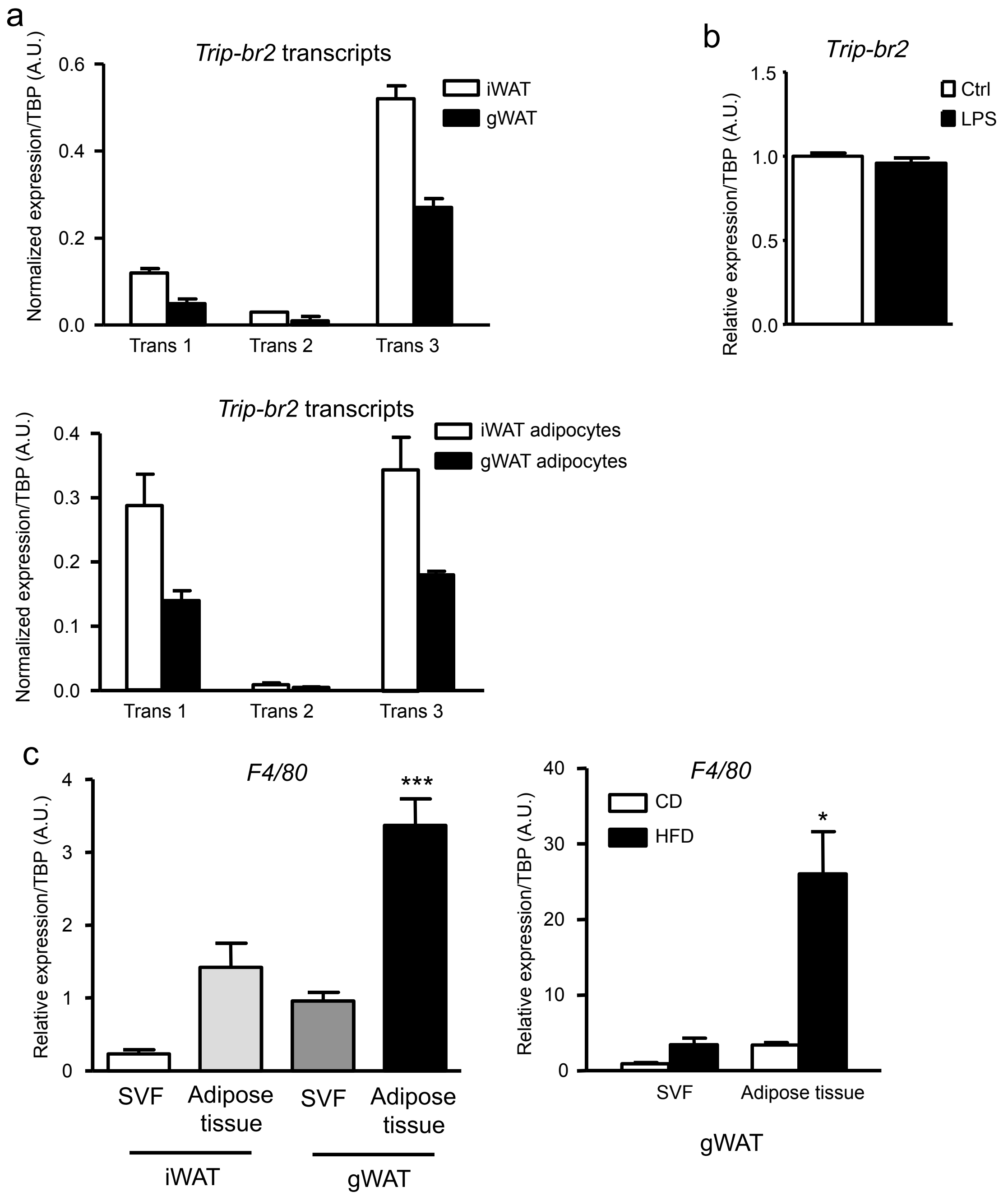

Supplementary Figure 1. TRIP-Br2 expression in WAT and adipocytes treated with LPS
(a) qPCR analysis of TRIP-Br2 transcripts gene expression in gonadal (gWAT) or inguinal (iWAT) adipose tissues or isolated mature adipocyted from chow diet-fed mice (n=6 per group replicated twice). (b) qPCR analysis of TRIP-Br2 gene expression in 3T3-L1 differentiated adipocytes treated with vehicle or LPS for 24 h (n=4 per group). (c) qPCR analysis of F4/80 gene expression in gWAT or iWAT stromal-vascular-fraction (SVF) or adipose tissues from CD or HFD-fed mice (n=5 per group replicated twice). All qPCR data are normalized with TBP and presented as mean ± SEM. Two-tailed student *t*-test, *,p<0.05; **,p<0.01; ***,p<0.001.

Supplementary Figure 2

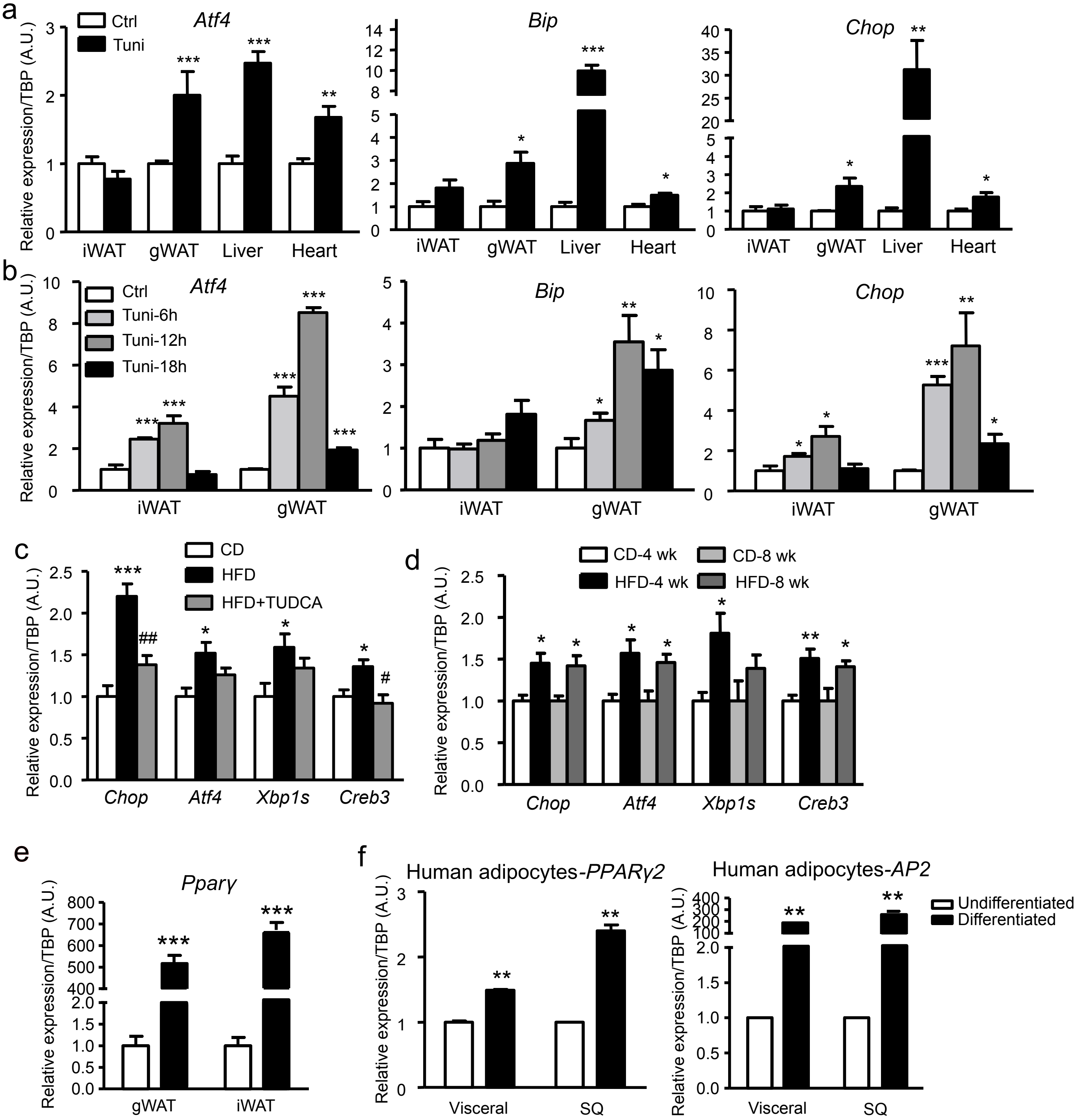

Supplementary Figure 2. ER stress markers induced by chemical or high-fat diet

qPCR analysis of ER stress markers gene expression in (a) tissues harvested from mice intraperitoneally (IP) injected with vehicle or tunicamycin (2.5 mg/kg) for 18 h (n=5 per group replicated thrice); (b) iWAT or gWAT from mice IP injected with vehicle or tunicamycin (2.5 mg/kg) for 6, 12 or 18 h (n=5 per group replicated twice); (c) gWAT from mice after 12 wk of CD, HFD or HFD with TUDCA (250 mg/kg at 8am and 8pm, i.p., total 500 mg/kg for 15 days) (n=5 per group); (d) gWAT from mice after 4 or 8 wk of CD or HFD (n=5 per group). qPCR analysis of PPARγ or AP2 gene expression in (e) undifferentiated or differentiated gWAT or iWAT preadipocytes or adipocytes (n=3 per group replicated twice); (f) undifferentiated or differentiated human visceral or subcutaneous preadipocytes or adipocytes (n=3 per group replicated twice). All qPCR data are normalized with TBP and presented as mean ± SEM. Two-tailed student *t*-test, *,p<0.05; **,p<0.01; ***,p<0.001 (vs CD); #, p<0.01; ##,p<0.01 (vs HFD).

Supplementary Figure 3

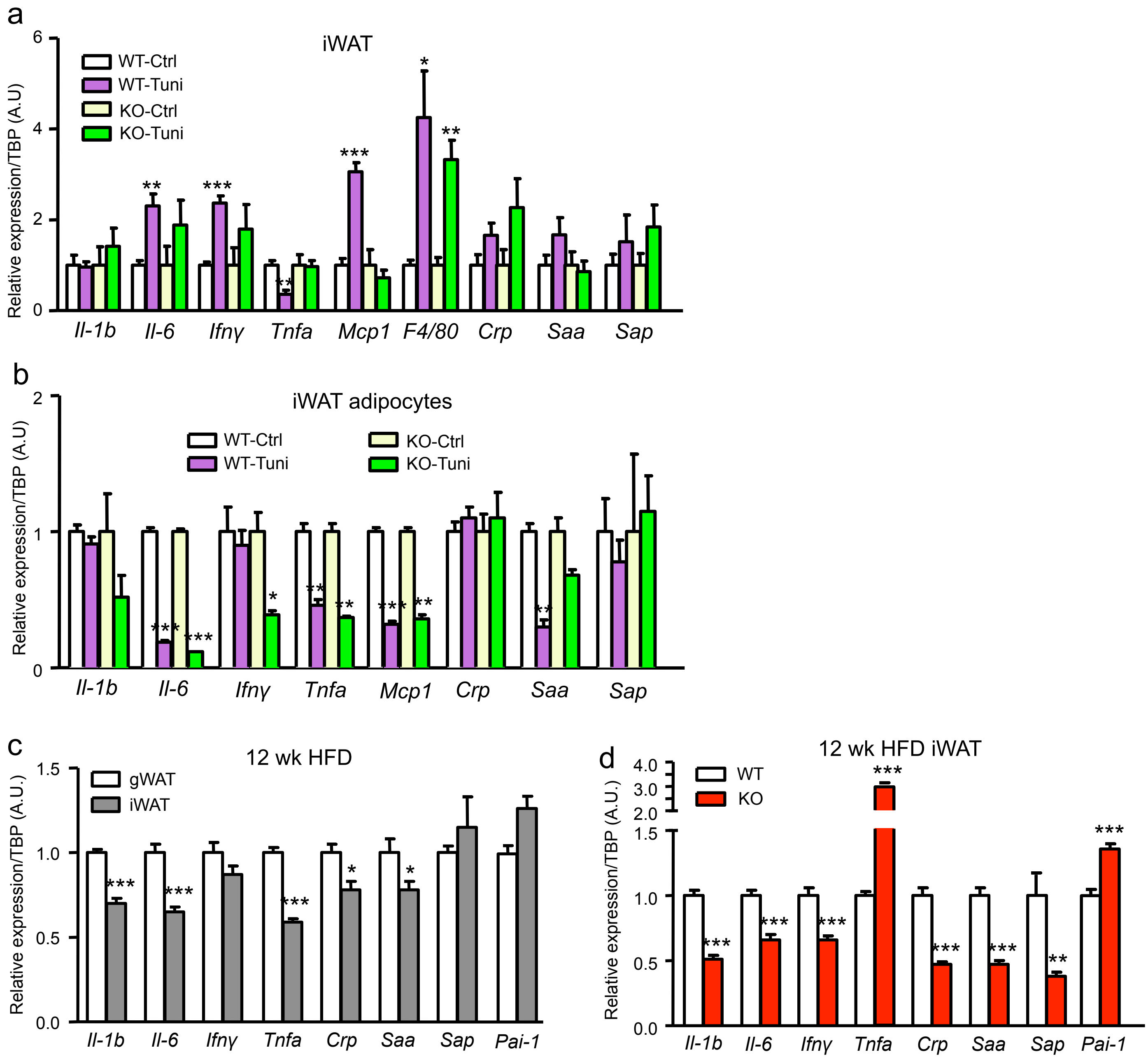

Supplementary Figure 3. Expression of inflammatory and acute phase response markers in WAT
qPCR analysis of inflammation and acute phase response markers gene expression in (a) iWAT from TRIP-Br2 WT or KO mice after 18 h of vehicle or tunicamycin (2.5 mg/kg, i.p.) treatment (n=5 per group replicated twice); (b) iWAT adipocytes differentiated from primary WT or KO SVF treated with or without tunicamycin (1 μg/ml) for 24 h (n=3 per group); (c) gWAT or iWAT from WT mice fed with 12 wk of HFD (n=6 per group replicated twice). (d) iWAT from WT or KO mice fed with 12 wk of HFD (n=6 per group replicated twice). All qPCR data are normalized with TBP and presented as mean ± SEM. Two-tailed student *t*-test, *,$p<0.05$; **,$p<0.01$; ***,$p<0.001$.

Supplementary Figure 4

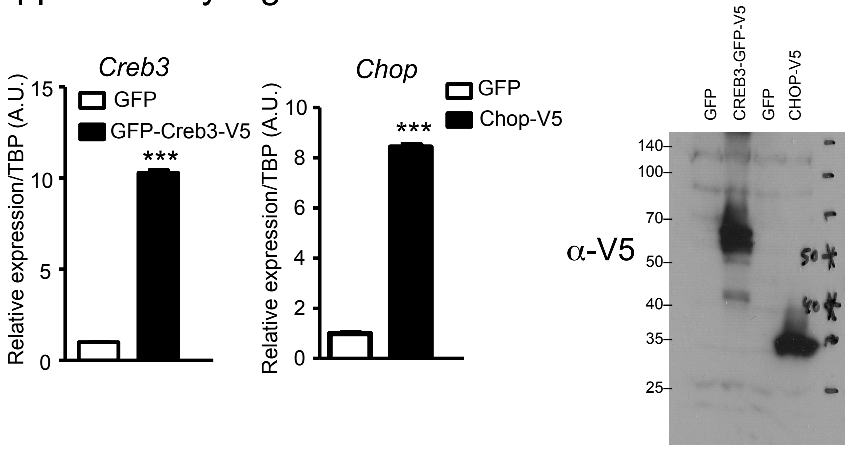

Supplementary Figure 4. Expression levels of CREB3 and CHOP

qPCR analysis of CREB3 and CHOP in adipocytes overexpressing GFP control, CREB-GFP-V5 or CHOP-V5 (n=3 per group). All qPCR data are normalized with TBP and presented as mean ± SEM. Two-tailed student *t*-test, ***,$p<0.001$; Western blot analysis for CREB or CHOP levels using anti-V5 antibody in adipocytes overexpressing GFP control, CREB-GFP-V5 or CHOP-V5.

Supplementary Figure 5

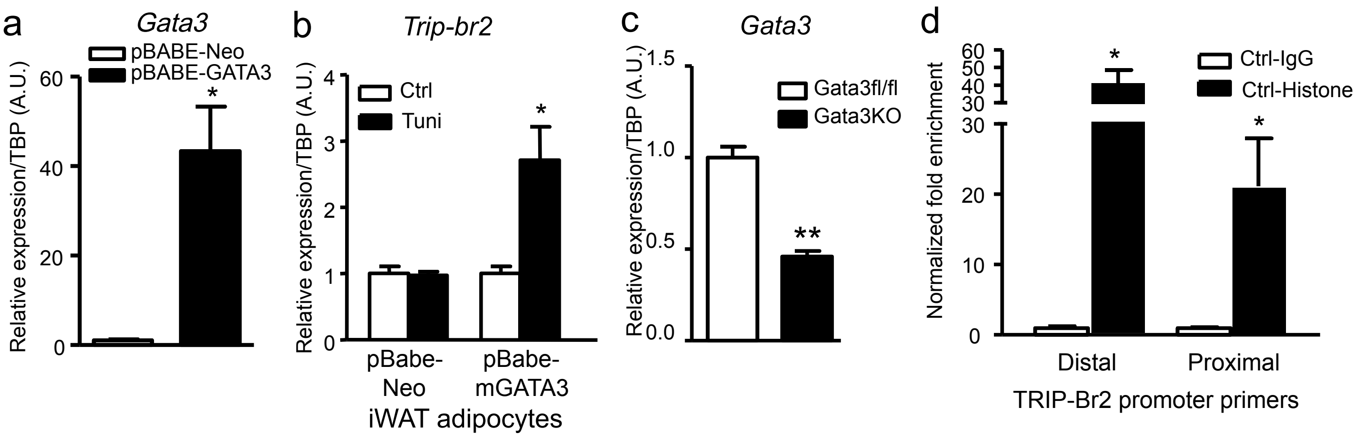

Supplementary Figure 5. Role of GATA3 on TRIP-Br2 expression

(a) qPCR analysis of GATA3 in gWAT adipocytes infected with control or GATA3 retrovirus (n=3 per group); (b) qPCR analysis of TRIP-Br2 in iWAT adipocytes infected with control or GATA3 retrovirus with or without tunicamycin treatment (n=3 per group). (c) qPCR analysis of GATA3 gene expression in gWAT adipocytes differentiated from GATA3fl/fl-CreERT2 primary SVF with or without tamoxifen (0.5 mM) treatment to induce GATA3 KO (n=5 per group). (d) qPCR analysis of proximal or distal genomic region of TRIP-Br2 transcript 3 promoter IgG (negative control) or anti-histone (positive control) immunoprecipitation (n=3 per group replicated twice). All qPCR data are normalized with TBP and presented as mean ± SEM. Two-tailed student *t*-test, *,p<0.05; **,p<0.01; ***,p<0.001.

Supplementary Figure 6

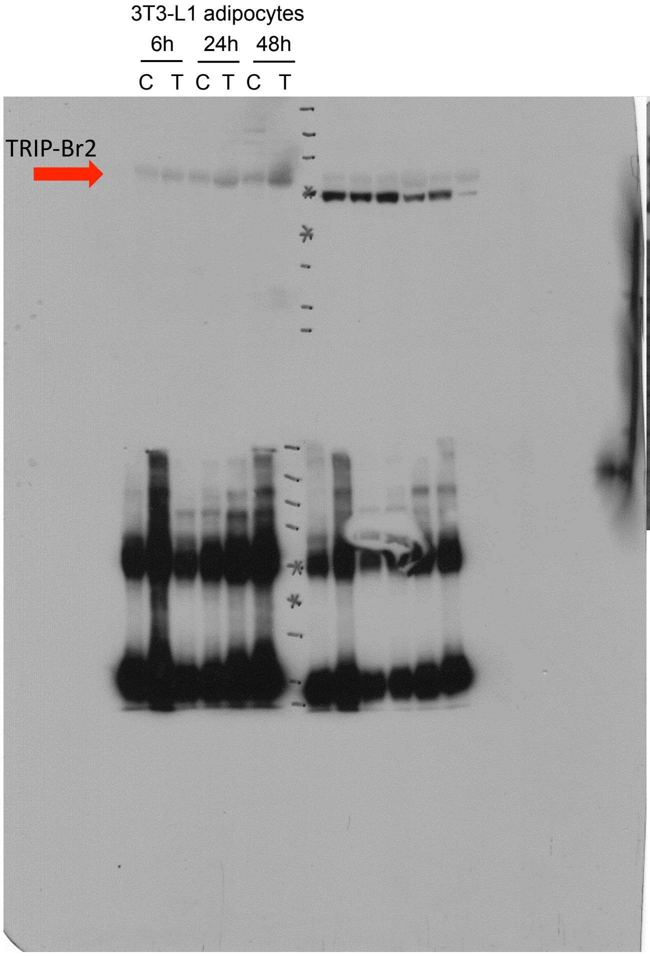

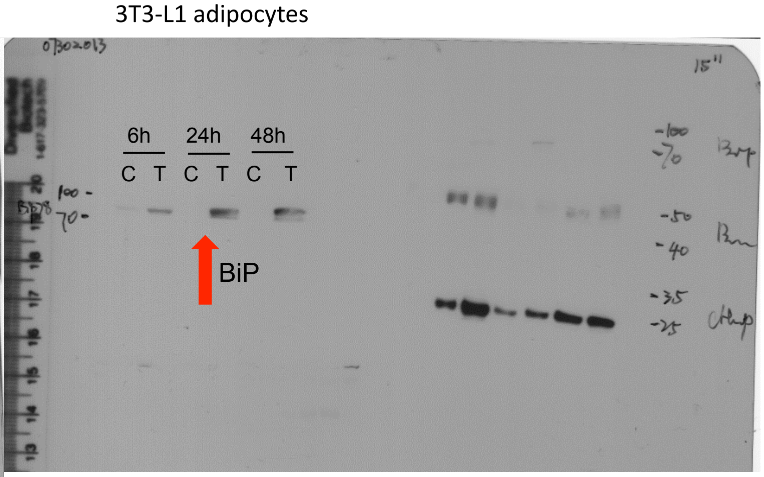

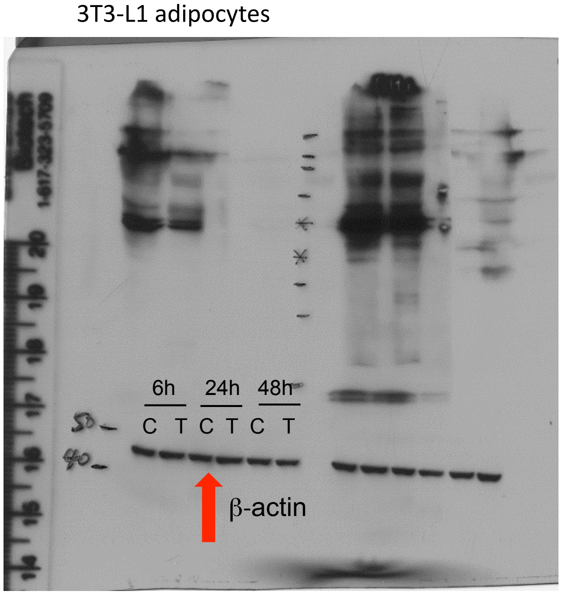

Supplementary Figure 6. Uncropped images of blots shown in Figure 2f

Supplementary Figure 7

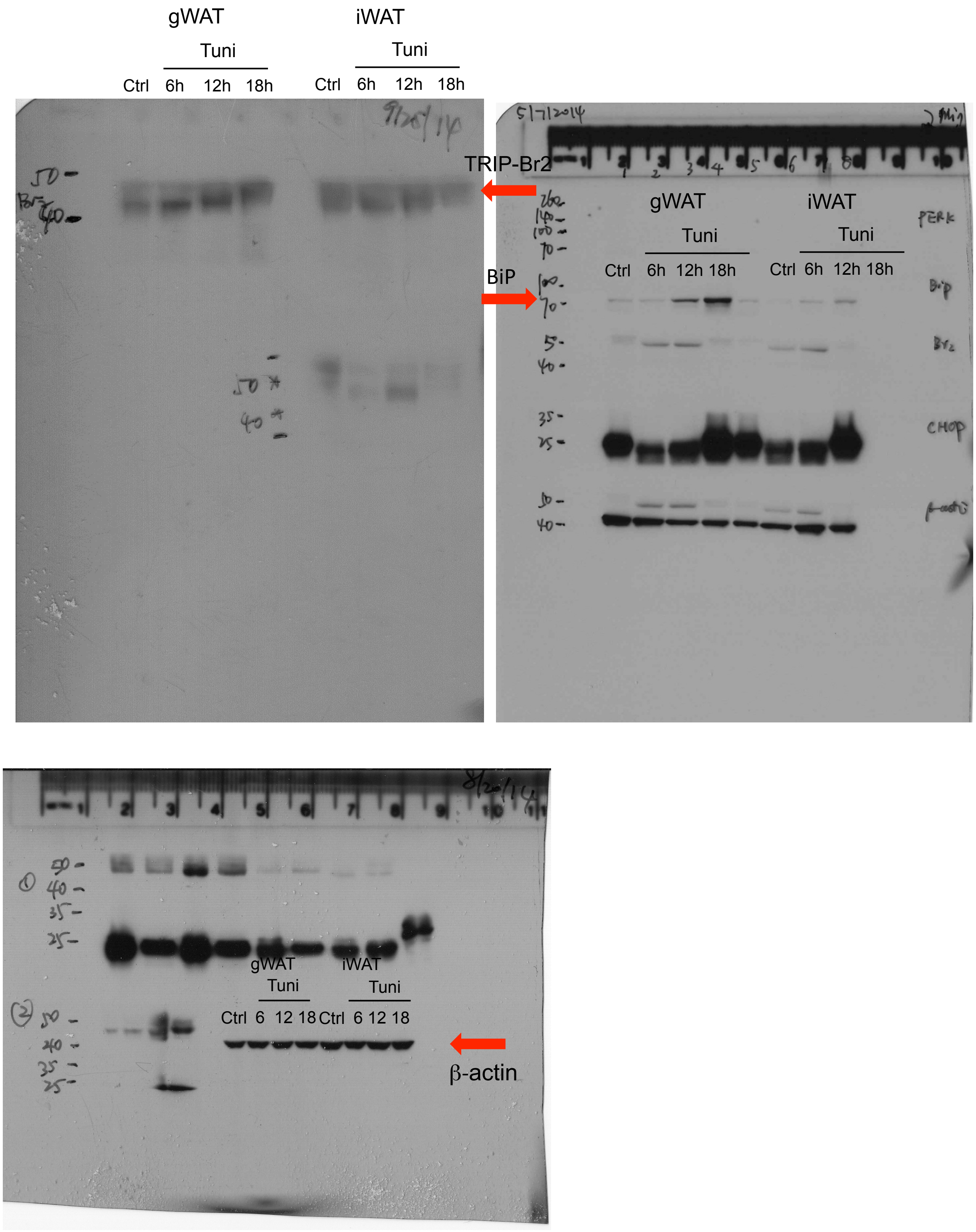

Supplementary Figure 7. Uncropped images of blots shown in Figure 3e

Supplementary Table 1: Compounds concentration for Figure 2a

Compound	Concentration
TNFa	100 ng/ml
MCP1	100 ng/ml
IFNg	10 μg/ml
IL6	2 μg/ml
IL10	100 ng/ml
BSA	0.5 M
Palmitate	0.2 M
Insulin	100 nM
IL1β	25 ng/ml
IL15	100 ng/ml
CRP	200 ng/ml
PAI-1	500 ng/ml
Leptin	100 ng/ml
Adiponectin	20 μg/ml

Supplementary Table 2.

Primer sequences used in this study

Gene		***Primer sequence***
mTrip-br2-tran1	Forward: 5'-	GCT CTC ACT CGT CGG GAT CC -3'
	Reverse: 5'-	TGG CGC TGT AAG GTG TAA GAC -3'
mTrip-br2-tran2	Forward: 5'-	GAA GGC TTT GTG CAC GTG AGG -3'
	Reverse: 5'-	TGG CGC TGT AAG GTG TAA GAC -3'
mTrip-br2-tran3	Forward: 5'-	GCT CCC TGC TAC CGT CGG C -3'
	Reverse: 5'-	TGG CGC TGT AAG GTG TAA GAC -3'
mTrip-br2-ORF	Forward: 5'-	ATA TAT GTT GGG TAA AGG AGG AA -3'
	Reverse: 5'-	TGG CGC TGT AAG GTG TAA GAC -3'
mIl-1b	Forward: 5'-	TGG AGA GTG TGG ATC CCA AGC AA T-3'
	Reverse: 5'-	TGT CCT GAC CAC TGT TGT TTC CCA -3'
mIl-6	Forward: 5'-	ATC CAG TTG CCT TCT TGG GAC TGA -3'
	Reverse: 5'-	TAA GCC TCC GAC TTG TGA AGT GGT -3'
mIfnγ	Forward: 5'-	TCT GGA GGA ACT GGC AAA AG -3'
	Reverse: 5'-	TTC AAG ACT TCA AAG AGT CTG AGG -3'
mMcp-1	Forward: 5'-	CCA CTC ACC TGC TGC TAC TCA T -3'
	Reverse: 5'-	TGG TGA TCC TCT TGT AGC TCT CC -3'
mPAI-1	Forward: 5'-	GGG ACG AAA CTG GAG ATG TTA T -3'
	Reverse: 5'-	GAG GAG TTG CCT TCT CTT TCT C -3'
mTnfa	Forward: 5'-	GCC TCT TCT CAT TCC TGC TTG T -3'
	Reverse: 5'-	GGC CAT TTG GGA ACT TCT CAT -3'
mCrp	Forward: 5'-	GGC GGG CAC TGA ACT ATA AA -3'
	Reverse: 5'-	CGA GAC AAG GGA GAG AAT GTA ATC -3'
mSaa1/2	Forward: 5'-	CTA GGA ACA CTG AAG ATG CTC TC -3'
	Reverse: 5'-	TCT CCT CCT CAA GCA GTT ACT A -3'
mSap	Forward: 5'-	TGT CTT CAC CAG CCT TCT TTC -3'
	Reverse: 5'-	TA GAT GTG GGA TCA GCT TCA C -3'
mF4/80	Forward: 5'-	TTT CCT CGC CTG CTT CTT C -3'
	Reverse: 5'-	CCC CGT CTC TGT ATT CAA C -3'
mBiP	Forward: 5'-	TTC AGC CAA TTA TCA GCA AAC TCT -3'
	Reverse: 5'-	TTT TCT GAT GTA TCC TCT TCA CCA GT -3'
mChop	Forward: 5'-	CCA CCA CAC CTG AAA GCA GAA -3'
	Reverse: 5'-	AGG TGA AAG GCA GGG ACT CA -3'
mXBP1s	Forward: 5'-	CTG AGT CCG AAT CAG GTG CAG -3'
	Reverse: 5'-	GTC CAT GGG AAG ATG TTC TGG -3'
mAtf4	Forward: 5'-	TTC CGG GAC AGA TTG GAT GTT GGA -3'
	Reverse: 5'-	ATG GCC AAT TGG GTT CAC TGT CTG -3'
mAtf6	Forward: 5'-	CTG GGC TCG GTA GTT TGT ATC -3'
	Reverse: 5'-	AGA CCT GAA TGG CTG CTT AC -3'
mCreb3	Forward: 5'-	CCA CTC TGG GAT CTT TCT TCA G -3'
	Reverse: 5'-	AAA GAA GGG CTG TGG TTA GG -3'
mGata1	Forward: 5'-	ACT AAC TGT CAA ACG ACC ACT AC-3'

	Reverse: 5'-	TCA CCT GAT GGA GCT TGA AAT AG -3'
mGata2	Forward: 5'-	GGA GAA AGG AGT AGG CAA GAA G -3'
	Reverse: 5'-	CCC AAG AAC ACA AAT AGC ACA C -3'
mGata3	Forward: 5'-	AGC TGC CAG ATA GCA TGA AG -3'
	Reverse: 5'-	TAG GGC GGA TAG GTG GTA AT -3'
mTbp	Forward: 5'-	ACC CTT CAC CAA TGA CTC CTA TG -3'
	Reverse: 5'-	ATG ATG ACT GCA GCA AAT CGC -3'
mPparγ	Forward: 5'-	TCG CTG ATG CAC TGC CTA TG
	Reverse: 5'-	GAG AGG TCC ACA GAG CTG ATT
human TBP	Forward: 5'-	TGA TGC CTT ATG GCA CTG GAC TCA-3'
	Reverse: 5'-	CTG CTG CCT TTG TTG CTC TTC CAA-3'
human TRIP-BR2-ORF	Forward: 5'-	GAC TCT CTG CCT GGG AAT TT -3'
	Reverse: 5'-	GGA CGT ATC AAT GTC AGC AAA C -3'
human GATA3	Forward: 5'-	CTC GGA GGG TTT CTT GTT TCT -3'
	Reverse: 5'-	CAG GAA TAG GGA CAA GAC AGA TG-3'
human BIP	Forward: 5'-	AGA GTG AGA CTC CAC CTC AA -3'
	Reverse: 5'-	CAG CCA GTT GCC CAT CTA TAA -3'
human PPARγ	Forward: 5'-	AGC CTC ATG AAG AGC CTT CCA
	Reverse: 5'-	TCC GGA AGA AAC CCT TGC A
human AP2	Forward: 5'-	CGT CAC TTC CAC GAG AGT TTA T
	Reverse: 5'-	TCC CAC AGA ATG TTG TAG AGT TC
